# Low-Complexity Soft-Output Signal Detection Based on Improved Kaczmarz Iteration Algorithm for Uplink Massive MIMO System

**DOI:** 10.3390/s20061564

**Published:** 2020-03-11

**Authors:** Hebiao Wu, Bin Shen, Shufeng Zhao, Peng Gong

**Affiliations:** 1 Chongqing Key Laboratory of Mobile Communications Technology, School of Communication and Information Engineering (SCIE), Chongqing University of Posts and Telecommunications (CQUPT), Chongqing 400065, China; s180131005@stu.cqupt.edu.cn (H.W.); shenbin@cqupt.edu.cn (B.S.); s170101160@stu.cqupt.edu.cn (S.Z.); 2 National Key Laboratory of Mechatronical Engineering and Control, School of Mechatronical Engineering, Beijing Institute of Technology (BIT), Beijing 100081, China

**Keywords:** massive MIMO, low-complexity, Kaczmarz iteration, relaxation parameter, soft output

## Abstract

For multi-user uplink massive multiple input multiple output (MIMO) systems, minimum mean square error (MMSE) criterion-based linear signal detection algorithm achieves nearly optimal performance, on condition that the number of antennas at the base station is asymptotically large. However, it involves prohibitively high complexity in matrix inversion when the number of users is getting large. A low-complexity soft-output signal detection algorithm based on improved Kaczmarz method is proposed in this paper, which circumvents the matrix inversion operation and thus reduces the complexity by an order of magnitude. Meanwhile, an optimal relaxation parameter is introduced to further accelerate the convergence speed of the proposed algorithm and two approximate methods of calculating the log-likelihood ratios (LLRs) for channel decoding are obtained as well. Analysis and simulations verify that the proposed algorithm outperforms various typical low-complexity signal detection algorithms. The proposed algorithm converges rapidly and achieves its performance quite close to that of the MMSE algorithm with only a small number of iterations.

## 1. Introduction

Massive multiple-input multiple-output (MIMO) technology dramatically expands the capacity of wireless communication systems without increasing the system bandwidth and transmit power and effectively resolves the contradiction between the limited spectrum resource and the rapid growth in capacity demand. Therefore, it has become one of the most promising solutions of 5G systems [1,2,3,4]. Equipped with up to hundreds of antennas at the base station (BS), massive MIMO system simultaneously serves multiple single-antenna users in the network, resulting in an order of magnitude improvement in spectrum utilization and energy efficiency of wireless systems [5,6].

Apart from the salient technical merits, massive MIMO system encounters various challenging problems in practice, one of which is the multi-user signal detection in the uplink. Due to the large number of antennas, the multi-user interference is remarkably intensified in system uplink and the implementation complexity is highly accrued, compared with the conventional MIMO systems. Theoretically, the maximum likelihood (ML) algorithm serves as the optimal solution for signal detection in MIMO systems and it becomes stringently burdensome to be implemented effectively in practical applications. Since the computational complexity of ML algorithm rises exponentially with the increase of the number of antennas and the modulation order of the baseband signal [6,7], it is actually infeasible to be employed in massive MIMO systems. When the reduced or fixed complexity is considered as the first priority design objective, the tabu search (TS) algorithm [8] and the fixed-complexity sphere decoding (FSD) algorithms [9] were proposed to obtain the close optimal ML detection performance, but their complexity still becomes not practically affordable for a large scale configuration of MIMO system with high modulation order.

Benefiting from the large number of antennas in massive MIMO systems, linear signal detection algorithms, such as zero forcing (ZF) and minimum mean square error (MMSE) algorithms, have been shown and verified to achieve nearly optimal detection performance, at the cost of involving high-dimensional matrix inversion with high complexity [6] ($O(K^{3})$, where *K* is the number of users simultaneously transmitting over the uplink). In recent years, various low complexity signal detection algorithms based on the MMSE criterion have been proposed for massive MIMO systems in the literature. In our previous work, we have investigated a variety of low complexity signal detection algorithms for massive MIMO systems under the MMSE criterion-based signal detection, where the key idea of achieving simplified complexity in signal detection is to find a solution that manages to evade the high-dimension matrix inverse operation, and a comparative study has been presented [10].

To the authors’ best knowledge, the MMSE criterion-based low complexity signal detection algorithms can be basically categorized into three typical types, namely the approximate matrix inversion algorithms (AMIA), the iterative approaches for solving linear equations (IASLE), and the matrix gradient search methods (MGSM). Firstly, the AMIA algorithms deal with the matrix inversion operation, required by the MMSE signal detection, in an approximation manner where the Neumann series expansion and Newton iteration are evoked to estimate the matrix inversion approximately [11,12,13]. The approximation accuracy depends on the number of Neumann items or the Newton iterations and may result in a high complexity when the number of items or iterations is set large for achieving a satisfactory performance. Secondly, in an entirely different mechanism, the IASLE tackle the problem of matrix inversion by finding solution to the system equation [14,15,16,17], where the transmitted multi-user signal vector is directly estimated and thus the high dimensional matrix inversion is purposely circumvented. Thirdly, based on the same idea of the IASLE, the MGSM methods are proposed to acquire the equation solution by matrix gradient search and hence the direct matrix inversion operations are bypassed, saving a lot of computations [18,19]. From the perspective of system performance, the AMIA algorithms are usually inferior to the IASLE and MGSM algorithms and when the number of items or iterations is large, the algorithm complexity is approaching $O(K^{3})$ again. As for the IASLE and MGSM algorithms, in case that some special properties of the weighting matrix is not guaranteed, for instance, if the weighting matrix is not symmetric positive and strictly diagonal dominant, they may encounter serious performance degradation or even fail to operate properly. Drawbacks of these algorithms need to be overcome by means of finding new type of algorithms. The aforementioned typical MMSE criterion-based multi-user signal detection algorithms are compared in Table 1.

In this paper, in order to obtain an easy-to-implement multi-user signal detection scheme for the uplink massive MIMO system, we propose a soft decision algorithm based on the Kaczmarz iteration [20,21,22]. In our previous work, the Kaczmarz algorithm was proposed to serve as a matrix-inverse approximation method for implementing the MMSE criterion-based signal detection with reduced complexity [22]. By circumventing the high-dimensional matrix inversion computations, we effectuated a simplified detection scheme for acquiring the transmitted signal vector in linear equation solving manner. To further improve the system performance and accelerate the converging speed in iterations, an optimal relaxation parameter is introduced to accelerate the convergence of the proposed Kaczmarz algorithm. To be more specific, the proposed improved Kaczmarz algorithm falls in one category of the low complexity MMSE criterion-based signal detection algorithms, that is, the IASLE, and it is actually a combination of the iterative approach for solving linear equations and the conventional Kaczmarz algorithm. Based on the output of the proposed improved Kaczmarz algorithm, theoretical log-likelihood ratios (LLRs) of the user bit streams are derived and one approximate method of estimating the LLRs for channel decoding is presented as well. Simulation results verify that the proposed algorithm outperforms the typical algorithms mentioned in Table 1 in terms of bit error rate (BER) with significantly relieved computational complexity. In comparison with the Kaczmarz algorithm, the proposed improved Kaczmarz algorithm yields a much better BER performance, given the same number of iterations. Additionally, the proposed improved Kaczmarz algorithm converges rapidly in operation and achieves its performance quite close to that of the MMSE algorithm with only a small number of iterations.

The rest of this paper is organized as follows. In Section 2, we describe the system model. In Section 3, the Kaczmarz iteration based signal detection is introduced. In Section 4, we propose the improved Kaczmarz algorithm based soft output signal detection. In Section 5, simulation and analysis are presented. Finally, Section 6 concludes the paper.

Notation: Lower-case and upper-case boldface letters are used to represent column vectors and matrices, respectively. The superscripts ${\left(\cdot \right)}^{T}$, ${\left(\cdot \right)}^{H}$, and ${\left(\cdot \right)}^{-1}$ stand for the transpose, conjugate-transpose, and inverse of matrix, separately. The operator $∥\cdot ∥$, E[·], and <·,·> denote the vector/matrix norm, the statistical expectation of a given argument, and the inner product of two vectors, respectively. $I_{K}$ is the *K* dimensional unit diagonal matrix.

## 2. System Model

An uplink massive MIMO system is considered, where the BS is equipped with *N* antennas and totally *K* single-antenna users are located within the coverage area of the BS (N≫K). The bit stream of each user is first encoded using a channel encoder and then mapped to the constellation points in the set C. The constellation symbol vector $s={\left[s_{1},s_{2},\cdots ,s_{K}\right]}^{T}$, $s\in C^{K}$, contains the transmit signals from the *K* users and it is assumed that $E\left\{{\left|s_{k}\right|}^{2}\right\}=E_{s},k\in \left\{1,2,\cdots ,K\right\}$, where $E_{s}$ represents the average power of the user signals.

Based on the system configuration, the received signal at the BS can be expressed as:

(1) y=Hs+n,

where $y={\left[y_{1},y_{2},\cdots ,y_{N}\right]}^{T}$ is the received signal vector at the BS, n is the white Gaussian noise vector with zero mean and variance $\sigma_{0}^{2}$ for each entry, and $H=[h_{1},h_{2},\cdots ,h_{K}]$ is the N×K channel matrix with its entry ${\left[H\right]}_{nk}$ denoting the channel coefficient between the *k*-th user and the *n*-th BS antenna.

### 2.1. MMSE Detection

If linear detection is utilized under the MMSE criterion, an estimate of the transmitted signal, $\hat{s}={\left[{\hat{s}}_{1},{\hat{s}}_{2},\cdots ,{\hat{s}}_{K}\right]}^{T}$, at the BS is expressed as:

(2) $$\hat{s}=Fy=W^{-1}\hat{y}={\left(G+\sigma_{0}^{2}I_{K}\right)}^{-1}H^{H}y,$$

where $F\;=\;W^{-1}H^{H}\;=\;{\left(G\;+\;\sigma_{0}^{2}I_{K}\right)}^{-1}H^{H}$ indicates the weighting matrix, $\hat{y}=H^{H}y$ is the matched filter output, and $G=H^{H}H$ is the K×K Gram matrix.

### 2.2. LLRs Generation

Based on the MMSE weighting, the transmitted signal can be presented as:

(3) $$\begin{array}{cc} \hat{s} & =W^{-1}Gs+W^{-1}H^{H}n\\ \; & =Us+W^{-1}H^{H}n, \end{array}$$

where U represents the channel matrix after equalization.

The received signal of the *k*-th user can be expressed as:

(4) $${\hat{s}}_{k}=\mu_{k}s_{k}+p_{k},$$

where $\mu_{k}\;=\;{\left[U\right]}_{kk}\;=\;U_{kk}$ is the equivalent channel gain after equalization and $p_{k}$ denotes the noise-plus interference (NPI) for the *k*-th user, with its variance calculated as:

(5) $$v_{k}^{2}=\sum_{j=1,j\neq k}^{K}{\left|U_{jk}\right|}^{2}+E_{kk}\sigma_{0}^{2},$$

where $E_{kk}$ is the *k*-th diagonal element of E, and $E=W^{-1}H^{H}{\left(W^{-1}H^{H}\right)}^{H}=W^{-1}GW^{-1}$.

The LLR of the *b*-th bit of the *k*-th user’s symbol is hence obtained as:

(6) $$L_{k,b}=Y_{k}\left(\min_{a\in C_{b}^{0}}{\left|\frac{{\hat{s}}_{k}}{\mu_{k}}-a\right|}^{2}-\min_{a^{\prime }\in C_{b}^{1}}{\left|\frac{{\hat{s}}_{k}}{\mu_{k}}-a^{\prime }\right|}^{2}\right),$$

where the coefficient $Y_{k}=\mu_{k}^{2}/v_{k}^{2}$ is equivalently the signal-to-interference-plus-noise ratio (SINR) for the *k*-th user, $C_{b}^{0}$ and $C_{b}^{1}$ are the symbol subsets of C where the *b*-th bit of the constellation symbols is 0 and 1, respectively.

From the above analysis, it is easy to observe that the calculations of F, U, and E all require computation of $W^{-1}$ first, leading to a high complexity as $O(K^{3})$. In order to circumvent the high complexity computations in matrix inversion, a low complexity MMSE soft detection scheme is proposed in this paper.

## 3. Kaczmarz Iteration Based Signal Detection

Since the IASLE methods usually outperform the AMIA algorithms, we also consider another typical IASLE algorithm, known as the Kaczmarz algorithm, in handling the signal detection task as the one of system equation solving. The Kaczmarz algorithm is widely used in various fields, among which it is also known as the algebraic reconstruction technique (ART) [23] in computed tomography. It provides an iterative method for solving the large scale over-determined linear equation Ax=b, where A is an N×K matrix (N≫K), x represents a K×1 vector to be determined, and b is an N×1 measurement vector. In the iterative process of the Kaczmarz algorithm, $a_{k}$, the *k*-th row of the matrix A, is traversed in a periodic manner. In each step, the solution of the last inner iteration is ${\hat{x}}_{t,k-1}$ within the *t*-th outer iteration and it is orthogonally projected, as $\langle a_{k}^{T},{\hat{x}}_{t,k-1}\rangle =b_{k}$, onto the hyperplane associated with the row vector $a_{k}$. Given an initial solution ${\hat{x}}_{0}$ for solving Ax=b, the *t*-th iteration’s solution of the Kaczmarz algorithm can be expressed as:

(7) $${\hat{x}}_{t,k}={\hat{x}}_{t,k-1}+\frac{b_{k}-\langle a_{k}^{T},{\hat{x}}_{t,k-1}\rangle }{∥a_{k}{∥}_{2}^{2}}a_{k}^{T},\;t=1,2,\cdots ,T_{\mathrm{Iter}};k=1,2,\cdots ,K$$

where *t* and *k* respectively represent the index of outer and inner iterations, $T_{\mathrm{Iter}}$ is the predetermined largest number of iterations, and ${∥a_{k}∥}_{2}$ is the vector norm of $a_{k}$.

Applying the Kaczmarz algorithm to detecting the transmitted signal $\hat{s}$ in the linear equation $W\hat{s}=\hat{y}$ for massive MIMO systems [22], we can obtain an estimate of the transmitted signal as:

(8) $${\hat{s}}_{t,k}={\hat{s}}_{t,k-1}+\frac{{\hat{y}}_{k}-\langle w_{k}^{T},{\hat{s}}_{t,k-1}\rangle }{∥w_{k}{∥}_{2}^{2}}w_{k}^{T},$$

where $w_{k}$ is the *k*-th row vector of W and the initial solution is denoted as ${\hat{s}}_{0}$ that is usually set as a zero vector. Details of the Kaczmarz algorithm based signal detection are given in Algorithm 1.
**Algorithm 1** Kaczmarz algorithm based signal detection**Input:**y, H, $\sigma_{0}^{2}$, $T_{\mathrm{Iter}}$.**Output:**${\hat{s}}_{T_{\mathrm{Iter}},K}$.  1: $\hat{y}=H^{H}y$, $W=H^{H}H+\sigma_{0}^{2}I_{K}$,  2: ${\hat{s}}_{0}=0$, t=0, k=1,  3: **for**
$t=1\;to\;T_{\mathrm{Iter}}$
**do**  4:     **for**
k=1toK
**do**  5:        ${\hat{s}}_{t,k}={\hat{s}}_{t,k-1}+\frac{{\hat{y}}_{k}-\langle w_{k}^{T},{\hat{s}}_{t,k-1}\rangle }{∥w_{k}{∥}_{2}^{2}}w_{k}^{T},$  6:        k=k+1,  7:      **end for**  8:    t=t+1.  9: **end for**

## 4. Improved Kaczmarz Algorithm Based Soft Output Signal Detection

As a non-optimal solution compared to the MMSE signal detection, the performance of the Kaczmarz algorithm can be ameliorated. We propose an improved Kaczmarz algorithm, utilizing a traversal scheme based on norm ordering to enhance the signal detection performance. Specifically, a traversal scheme based on norm ordering consists of the following three steps.

Step-1: *Norm ordering*. The norms of ${∥w_{k}∥}_{2}$ are sorted in descending order, resulting in the corresponding subscript set $\psi =\{\kappa_{1},\kappa_{2},\cdots ,\kappa_{K}\}$ permutated from the index set {1,2⋯,K}, with $\psi \left(k\right)=\kappa_{k}$, and then the entry in the subscript set ψ is sequentially selected for traversing. Equation (8) can be expressed as:

(9) $${\hat{s}}_{t,k}={\hat{s}}_{t,k-1}+\frac{{\hat{y}}_{\psi (k)}-\langle w_{\psi (k)}^{T},{\hat{s}}_{t,k-1}\rangle }{∥w_{\psi (k)}{∥}_{2}^{2}}w_{\psi (k)}^{T}.$$

Step-2: *Introducing the relaxation parameter in iterations*. An optimal relaxation parameter is introduced to accelerate convergence. Equation (9) can be modified as:

(10) $${\hat{s}}_{t,k}={\hat{s}}_{t,k-1}+\omega_{t,k}\frac{{\hat{y}}_{\psi (k)}-\langle w_{\psi (k)}^{T},{\hat{s}}_{t,k-1}\rangle }{∥w_{\psi (k)}{∥}_{2}^{2}}w_{\psi (k)}^{T},$$

where $\omega_{t,k}$ denotes the relaxation parameter for the (t,k)-th iteration.

Step-3: *Finding the optimal relaxation parameter*. The convergence speed of the Kaczmarz Algorithm merely depends on the condition number of W and the algorithm converges for any *t* and *k* when $\omega_{t,k}$ is set as a constant ω∈(0,2) [24]. Equation (10) can be simply expressed as:

(11) $${\hat{s}}_{t,k}={\hat{s}}_{t,k-1}+\omega \frac{{\hat{y}}_{\psi (k)}-\langle w_{\psi (k)}^{T},{\hat{s}}_{t,k-1}\rangle }{∥w_{\psi (k)}{∥}_{2}^{2}}w_{\psi (k)}^{T}.$$

Usually, the relaxation parameter ω is set as 1 for simplicity, as shown in Equation (9). However, it is heuristically found in experiments that the optimal relaxation parameter $\omega_{\mathrm{opt}}$ can be set as $\left(1+\lambda_{\min}/\lambda_{\max}\right)$, where $\lambda_{\min}$ and $\lambda_{\max}$ is the minimum and maximum eigenvalue of W, respectively.

In massive MIMO uplink system, as the number of BS antennas and the number of users increase, the eigenvalues of W obey the Marchenko-Pastur distribution, where $\lambda_{\min}$ and $\lambda_{\max}$ asymptotically converge as [2]:

(12) $$\begin{array}{cc} \; & \lambda_{\min}\to N{\left(1-\frac{1}{\sqrt{\alpha }}\right)}^{2},\\ \; & \lambda_{\max}\to N{\left(1+\frac{1}{\sqrt{\alpha }}\right)}^{2}, \end{array}$$

where α=N/K. The optimal relaxation parameter, as a function of α, can be therefore determined as:

(13) $$\omega_{\mathrm{opt}}=\lim_{N\to \infty ,K\to \infty }\left(1+\frac{\lambda_{\min}}{\lambda_{\max}}\right)=2-\frac{4\sqrt{\alpha }}{1+\alpha +2\sqrt{\alpha }},$$

where it is easy to observe the fact that $\omega_{\mathrm{opt}}\in \left(0,2\right)$. Based on the above analysis, we further demonstrate via simulations that the optimal relaxation parameter $\omega_{\mathrm{opt}}$ exists in a very narrow range and it can be set as a constant determined by the system configuration only. Detailed description of the improved Kaczmarz algorithm based soft output signal detection is presented in Algorithm 2.
**Algorithm 2** Improved Kaczmarz algorithm based soft output signal detection**Input:**y, H, $\sigma_{0}^{2}$, $T_{\mathrm{Iter}}$.**Output:**$L_{k,b}$.  1:% **Initialization**  2: $\hat{y}=H^{H}y$, $W=G+\sigma_{0}^{2}I_{K}$,  3: ${\hat{s}}_{0}=D^{-1}\hat{y}\approx \frac{1}{N}I_{K}\hat{y}$,  4: ${\hat{W}}_{0,0}^{-1}=D^{-1}$,  5: % **Setting the optimal relaxation parameter**  6: $\omega_{\mathrm{opt}}=2-\frac{4\sqrt{\alpha }}{1+\alpha +2\sqrt{\alpha }}$,  7: sort ${∥w_{k}∥}_{2}^{2}$ in descending order, obtaining the subscript set $\psi =\{\kappa_{1},\kappa_{2},\cdots ,\kappa_{K}\}$, $\psi \left(k\right)=\kappa_{k}$,  8: % **Solving the system equation in iterations**  9: **for**
$t=1\;\;to\;\;\;T_{\mathrm{Iter}}$
**do**  10:      **for**
k=1toK
**do**  11:         ${\hat{s}}_{t,k}={\hat{s}}_{t,k-1}+\omega \frac{{\hat{y}}_{\psi (k)}-\langle w_{\psi (k)}^{T},{\hat{s}}_{t,k-1}\rangle }{∥w_{\psi (k)}{∥}_{2}^{2}}w_{\psi (k)}^{T},$  12:         ${\hat{w}}_{\psi (k),t,k}^{-1}={\hat{w}}_{\psi (k),t,k-1}^{-1}$  13:              
$+\;\omega \frac{e_{\psi (k)}^{T}-w_{\psi (k)}{\hat{W}}_{t,k-1}^{-1}}{∥w_{\psi (k)}{∥}_{2}^{2}}W_{\psi (k)\psi (k)},$  14:         k=k+1,  15:      **end for**  16:     t=t+1.  17: **end for**  18: % **Computing the approximate LLRs**  19:
$\hat{U}={\hat{W}}_{T_{\mathrm{Iter}},K}^{-1}G,\hat{E}={\hat{W}}_{T_{\mathrm{Iter}},K}^{-1}G{\hat{W}}_{T_{\mathrm{Iter}},K}^{-1}=\hat{U}{\hat{W}}_{T_{\mathrm{Iter}},K}^{-1},$  20: $Y_{k}={\hat{\mu }}_{k}^{2}/{\hat{v}}_{k}^{2}$,  21: ${\hat{\mu }}_{k}={\hat{U}}_{kk}$,  22:
${\hat{v}}_{k}^{2}=\sum_{j\neq k,j=1}^{K}{\left|{\hat{U}}_{jk}\right|}^{2}+{\hat{E}}_{kk}\sigma_{0}^{2},$  23:
$L_{k,b}=Y_{k}\left(\min_{a\in C_{b}^{0}}{\left|\frac{{\hat{s}}_{k,T_{\mathrm{Iter}},K}}{{\hat{\mu }}_{k}}-a\right|}^{2}-\min_{a^{\prime }\in C_{b}^{1}}{\left|\frac{{\hat{s}}_{k,T_{\mathrm{Iter}},K}}{{\hat{\mu }}_{k}}-a^{\prime }\right|}^{2}\right).$

### 4.1. Initial Estimation

For massive MIMO systems, the columns of H are asymptotically orthogonal, meaning that $G=H^{H}H$ is positive definite and diagonally dominant. According to the channel hardening phenomenon [25], there is $G\approx NI_{K}$. This special property enables us to utilize $D^{-1}$ to approximate $W^{-1}$ with trivial error and the initial solution of Equation (11) can be set as:

(14) $${\hat{s}}_{0}=D^{-1}\hat{y}\approx \frac{1}{N}I_{K}\hat{y},$$

where D is the diagonal matrix corresponding to W.

### 4.2. Two Methods to Compute LLRs

#### 4.2.1. Exact Method

Through iterative operation, the solution vector ${\hat{s}}_{t,k}$ gradually converges to the solution for the equation $W\hat{s}=\hat{y}$, and hence there should be a corresponding matrix ${\hat{W}}_{t,k}^{-1}$ for each iteration. The most straightforward method of computing LLR is to use the Kaczmarz algorithm to estimate ${\hat{W}}_{t,k}^{-1}$ after each iteration. Combining Equations (2) and (11), an estimate of the transmit vector at each iteration can then be computed as:

(15) $$\begin{array}{cc} {\hat{s}}_{t,k} & ={\hat{W}}_{t,k}^{-1}\hat{y}\\ \; & ={\hat{W}}_{t,k-1}^{-1}\hat{y}+\omega_{\mathrm{opt}}\frac{e_{\psi (k)}^{T}\hat{y}-w_{\psi (k)}{\hat{W}}_{t,k-1}^{-1}\hat{y}}{∥w_{\psi (k)}{∥}_{2}^{2}}w_{\psi (k)}^{T}, \end{array}$$

where $e_{\psi (k)}^{T}$ represents the ψ(k)-th unit row vector that only has the ψ(k)-th element being non-zero.

For the ψ(k)-th element in ${\hat{s}}_{t,k}$, we have

(16) $$\begin{array}{cc} {\hat{s}}_{\psi (k),t,k} & ={\hat{w}}_{\psi (k),t,k}\hat{y}\\ \; & ={\hat{w}}_{\psi (k),t,k\;-\;1}\hat{y}+\omega_{\mathrm{opt}}\frac{e_{\psi (k)}^{T}\hat{y}-w_{\psi (k)}{\hat{W}}_{t,k\;-\;1}^{-1}\hat{y}}{∥w_{\psi (k)}{∥}_{2}^{2}}e_{\psi (k)}^{T}\;w_{\psi (k)}^{T}, \end{array}$$

where the corresponding ψ(k)-th row vector of ${\hat{W}}_{t,k}^{-1}$ can be expressed as:

(17) $${\hat{w}}_{\psi (k),t,k}^{-1}={\hat{w}}_{\psi (k),t,k-1}^{-1}+\omega_{\mathrm{opt}}\frac{e_{\psi (k)}^{T}-w_{\psi (k)}{\hat{W}}_{t,k-1}^{-1}}{∥w_{\psi (k)}{∥}_{2}^{2}}W_{\psi (k)\psi (k)},$$

where $W_{\psi (k)\psi (k)}$ is the ψ(k)-th diagonal element of W. Therefore, ${\hat{W}}_{t,k}^{-1}$ can be obtained from the above discussions, with an initial solution ${\hat{W}}_{0,0}^{-1}\;\;\;=\;\;\;D^{-1}$. The corresponding channel gain and NPI variance can be derived as:

(18) $${\hat{\mu }}_{k}^{\left(t\right)}={\hat{U}}_{kk}^{\left(t\right)},$$

(19) $${\left({\hat{v}}_{k}^{\left(t\right)}\right)}^{2}=\sum_{j\neq k,j=1}^{K}{\left|{\hat{U}}_{jk}^{\left(t\right)}\right|}^{2}+{\hat{E}}_{kk}^{\left(t\right)}\sigma_{0}^{2},$$

where ${\hat{U}}^{\left(t\right)}={\hat{W}}_{t,k}^{-1}G$ and ${\hat{E}}^{\left(t\right)}={\hat{W}}_{t,k}^{-1}G{\hat{W}}_{t,k}^{-1}={\hat{U}}^{\left(t\right)}{\hat{W}}_{t,k}^{-1}.$ Then, the LLRs for channel decoding can be obtained by substituting Equations (18) and (19) into Equation (6).

#### 4.2.2. Approximated Method

The exact method precisely computes the LLRs and yields theoretically optimal BER performance. However, from Equation (17), updating ${\hat{W}}_{t,k}^{-1}$ involves multiplication and addition between the matrix and the vector for each iteration, causing the final complexity order to rise to $O(K^{3})$ again. To solve this problem, an approximated method to calculate the LLRs is proposed, which completely avoids the complicated matrix inversion. Since $W^{-1}$ is diagonal dominant for uplink massive MIMO systems, it can be replaced by the diagonal matrix $D^{-1}$ with tolerable error. Then, the approximated channel gain and NPI variance are obtained in a non-iterative manner as:

(20) $${\tilde{\mu }}_{k}={\tilde{U}}_{kk},$$

(21) $${\tilde{v}}_{k}^{2}=\sum_{j\neq k,j=1}^{K}{\left|{\tilde{U}}_{jk}\right|}^{2}+{\tilde{E}}_{kk}\sigma_{0}^{2},$$

where $\tilde{U}=D^{-1}G$, $\tilde{E}=D^{-1}GD^{-1}=\tilde{U}D^{-1}.$ Then, the LLRs for channel decoding can be obtained by substituting Equations (20) and (21) into Equation (6) with much lower complexity.

## 5. Simulation and Analysis

### 5.1. Computational Complexity

The computational complexity is analyzed in terms of the number of real-valued multiplications. Since all algorithms mentioned in this paper need to calculate the filter matrix W and the matched filter output $\hat{y}$, and the LLR is computed via the proposed approximate method, only the computational complexity of other different implementation parts of each algorithm is analyzed and compared. The computational complexity of the improved Kaczmarz algorithm mainly comes from iteratively estimating $\hat{s}$. For each inner iteration, the calculation of the inner product of the two vectors in Equation (10) and the update of ${\hat{s}}_{t,k}$, require 2K real multiplication operations respectively. With the additional operations in updating the intermediate values, it totally requires $2K\left(4K+2\right)=8K^{2}\;+\;4K$ real multiplication operations for each outer iteration. The computational complexity of the algorithms investigated in the computer simulations can be found in Reference [10].

**Figure 1:**
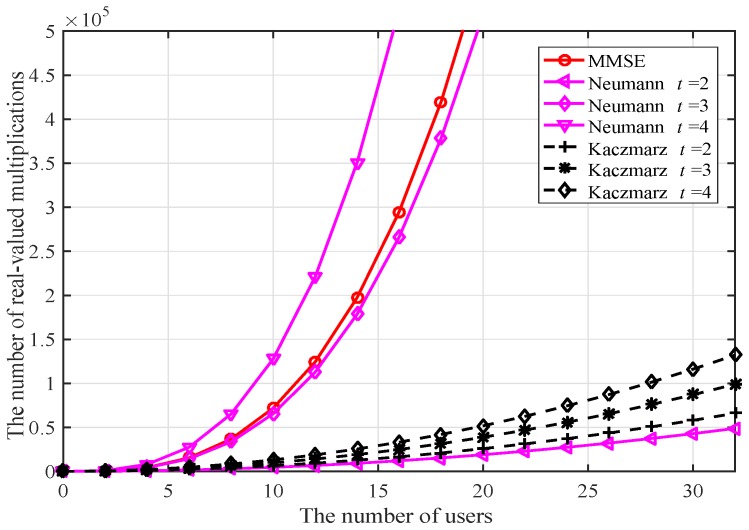
Algorithms complexity comparison.

**Figure 2:**
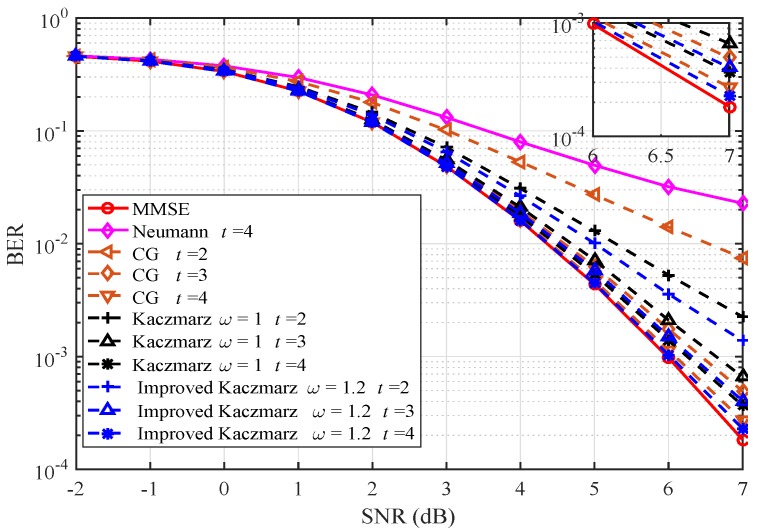
Bit error rate (BER) performance comparison (64×16).

Figure 1 shows the comparison of computational complexity among the MMSE, Neumann [12], and Kaczmarz algorithms, where the Kaczmarz algorithm demands significantly less than the other two algorithms when the number of iterations is small. Although the Neumann series expansion algorithm requires a slightly lower complexity than the Kaczmarz algorithm when its number of expansion items is two, the system BER performance of the Neumann algorithm is far from being satisfactory in practice, as shown in Figure 2. Moreover, when the number of expansion items is four, the complexity of the Neumann algorithm even surpasses that of the MMSE algorithm.

### 5.2. BER Performance

In Monte-Carlo simulations, Rayleigh fading channels are assumed. The channel coding scheme is implemented with convolutional coding with a code rate of 1/2 and the 16-QAM constellation is chosen for modulation. The average transmit power of each user is set as 1. The parameter *t* represents both the number of iterations and the number of expansion items of the Neumann algorithm.

**Figure 3:**
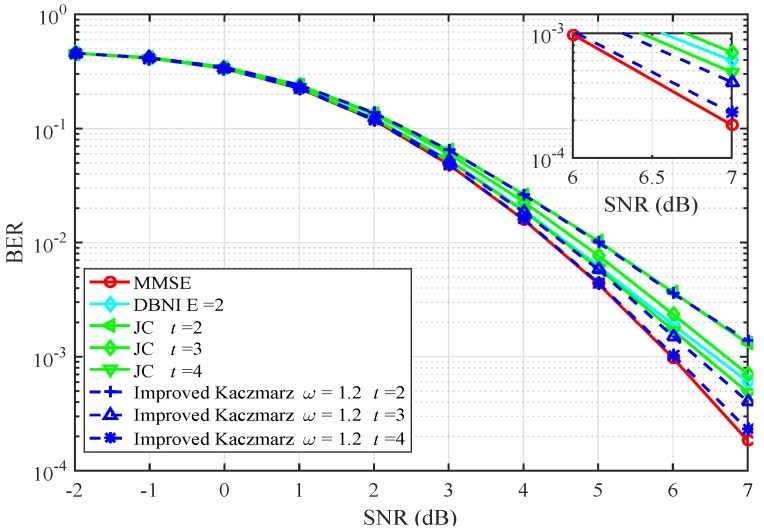
BER performance comparison (64×16).

Figure 2 and Figure 3 give the BER performance of the algorithms mentioned in this paper when the system antenna configuration (N,K) is (64,16). In Figure 2, the BER performance of Neumann [12], conjugate gradient (CG) [18], Kaczmarz, and the improved Kaczmarz algorithm is presented. The BER performance of the improved Kaczmarz algorithm is obtained with approximated LLR computations. It is obvious that the improved Kaczmarz algorithm performs best among all the low complexity signal detection algorithms, given the same number of iterations or expansion items. In Figure 3, diagonal band Newton iteration (DBNI) with the band width as E=2 [13], joint steepest descent and Jacobi method (JC) [16], and the improved Kaczmarz algorithm are compared. As expected, the improved Kaczmarz algorithm outperforms all the other algorithms with the same number of iterations and its performance is sufficiently close to that of the conventional MMSE algorithm.

**Figure 4:**
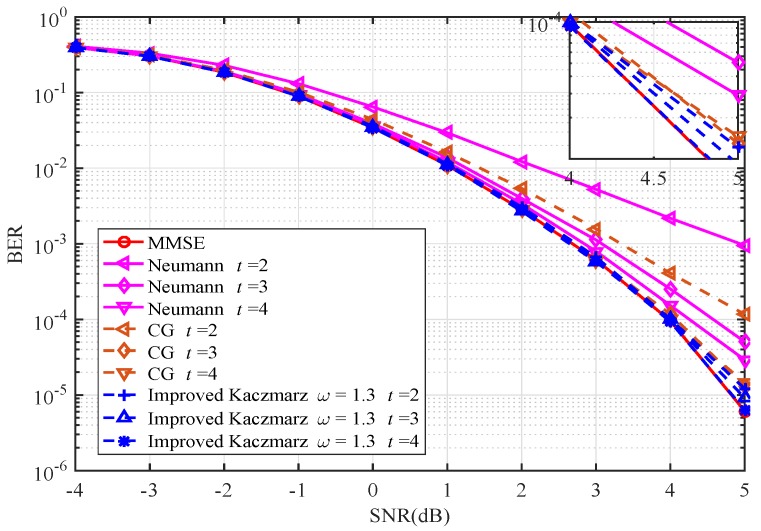
BER performance comparison (128×16).

**Figure 5:**
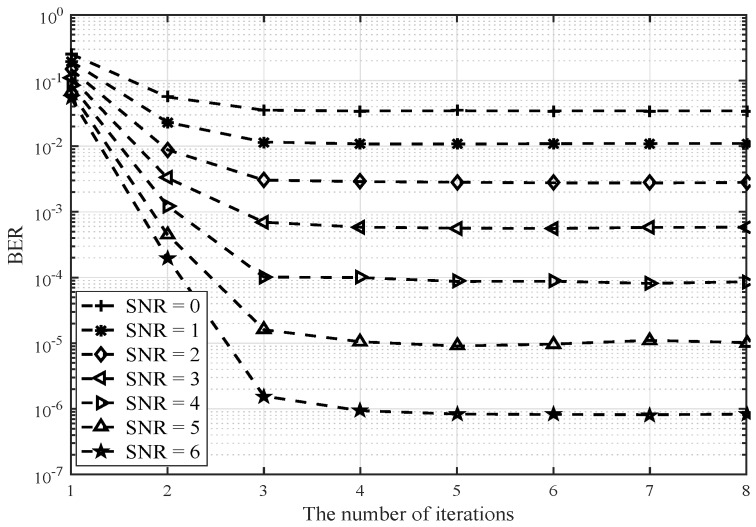
BER of the improved Kaczmarz algorithm with different number of iterations and SNR (128×16).

Figure 4 and Figure 5 illustrate the BER performance when the antenna configuration is (128,16). In Figure 4, the improved Kaczmarz algorithm is again verified to be superior to the other typical low complexity signal detection algorithms. Meanwhile, it is apparent that as the ratio K/N decreases from (64,16) in Figure 2 and Figure 3 to (128,16) in Figure 4 and Figure 5, the improved Kaczmarz algorithm approaches the performance of the MMSE algorithm faster and closer. In Figure 5, BER performance of the improved Kaczmarz algorithm with different numbers of iterations is provided. For different SNR, the performance is becoming stable after only three to four iterations.

**Figure 6:**
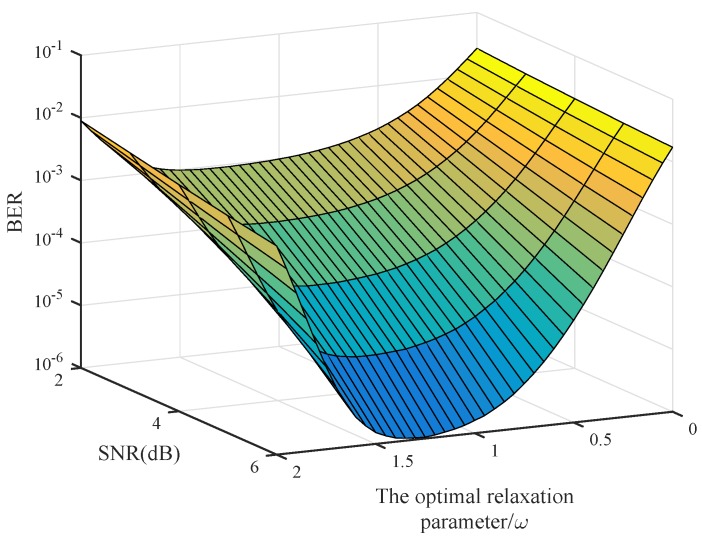
Impact of the relaxation parameter on BER (128×16).

In order to find the optimal relaxation parameter $\omega_{\mathrm{opt}}$, the impact of different relaxation parameters on the BER performance is provided in Figure 6. The antenna configuration is (128,16) and t=4. For different SNR, the optimal relaxation parameter $\omega_{\mathrm{opt}}$ is found to exist between 1.2 and 1.35. It is shown to be independent of the SNR and can be set as a constant.

## 6. Conclusions

In this paper, a low complexity soft output signal detection algorithm based on Kaczmarz iteration is proposed. The algorithm is tailored for uplink massive MIMO system to avoid high-dimensional matrix inversion required by the MMSE criterion. The improved Kaczmarz algorithm estimates the transmitted signal by iteratively solving the linear equation and circumventing the matrix inverse operation. Therefore, the complexity is significantly reduced from $O(K^{3})$ to $O(K^{2})$. Meanwhile, an optimal relaxation parameter is introduced to the improved Kaczmarz algorithm to further accelerate the algorithm convergence and enhance the BER performance. Simulation results verify that the proposed algorithm outperforms various conventional signal detection algorithms with approximate matrix inversion in terms of BER and computational complexity. The improved Kaczmarz algorithm converges rapidly and achieves its performance quite close to that of the MMSE algorithm with only a small number of iterations, and the complexity order remains as $O(K^{2})$ at any number of iterations. The algorithm can serve as a low-complexity candidate scheme for signal detection of the uplink massive MIMO system. 

## Figures and Tables

**Table 1:** Minimum mean square error (MMSE) criterion-based low complexity signal detection algorithms.

Category	Algorithm	Comparisons
AMIA	Neumann series approximation [12]	Relatively poor/unsatisfactory performance; High complexity required for large number of Neumann items or Newton iterations.
	Newton iteration [13]	
IASLE	Richardson [14]	Direct estimation of the transmitted signal vector via equation solving; Symmetric positive definite property demanded for the filtering matrix; Characteristics of being diagonally dominant required for the filtering matrix; Failure or performance deteriorated when above conditions not guaranteed.
	Gauss-Seidel [15]	
	Jacobi [16]	
	Successive over-relaxation [17]	
MGSM	Conjugate gradient [18]	Relatively high complexity in gradient update; Same requirements on the filtering matrix as that of the iterative methods.
	Steepest descent [19]	
